# Activation of the TLR1/2 pathway induces the shaping of the immune response status of peripheral blood leukocytes

**DOI:** 10.3892/etm.2014.1621

**Published:** 2014-03-14

**Authors:** YING PENG, LI ZHANG

**Affiliations:** 1 Department of Cardiology, West China Hospital, Sichuan University, Chengdu, Sichuan 610041, P.R. China; 2 Laboratory of Pathology, Department of Pathology, West China Hospital, Sichuan University, Chengdu, Sichuan 610041, P.R. China

**Keywords:** Toll-like receptor 1/2, agonist, peripheral blood leukocyte, immunomodulatory molecules

## Abstract

Toll-like receptors (TLRs) play an essential role in the activation and regulation of the innate and adaptive immune responses through the recognition of specific components of pathogens. TLR1/2 on the cell surface plays an important role in defending against Gram-positive bacteria. The aim of the present study was to examine the expressional variation of immunomodulatory molecules in peripheral blood leukocytes (PBLs) treated with the TLR1/2 agonist, Pam3Cys. The quantitative polymerase chain reaction result showed dramatically increased expression of immune-related factors treated with Pam3Cys. Antibody-chip assays confirmed that activation of TLR1/2 could induce secretion of four important immune factors [interleukin (IL)-6, IL-8, macrophage inflammatory protein-1α and interferon-β). Western-blot analysis indicated the upregulation of three significant signal kinase proteins (phosphorylated signal transducer and activator of transcription 3, extracellular signal-related kinase and c-Jun N-terminal kinase 2). The study demonstrated that there were numerous molecules involved in the immune response of PBLs stimulated by the TLR1/2 ligand. Our future studies will focus on the mechanisms of these molecules in the TLR1/2 agonist-mediated immune response.

## Introduction

Toll-like receptors (TLRs) are key regulators of the innate and adaptive immune responses, and are activated by specific pathogen-associated molecular patterns (1). The activation of TLRs initiates a signaling cascade and induces the expression of various important pro-inflammatory cytokines and finally leads to the eradication of infecting microbes (2).

The expression of TLR has been shown for several immune cells, whereby the amount of expression and the combination of TLR differs from one type to another (3). In a human peripheral blood mononuclear cell (PBMC) survey, TLR1 was expressed in all cell types examined, while high expression of TLR2 was characteristic for monocytes (4). Among all TLR members, TLR1 usually interacts physically and functionally with TLR2, which appears to be involved in the discrimination of subtle changes in the lipid portion of lipoproteins (5).

The present study examined the *in vitro* responsiveness of PBLs from normal healthy volunteers to the TLR1/2 agonist in order to determine which types of immunomodulatory molecules are involved in the activation of the TLR1/2 pathway and in the promotion of the inflammatory status of PBLs.

## Materials and methods

### Isolation and stimulation of PBLs

Mixed PBLs were isolated from the blood of normal healthy volunteers using gradient centrifugation (800 × g for 20 min; Sigma-Aldrich, Oakville, ON, Canada), according to the manufacturers’ protocol. The PBLs (2×10^6^) were treated with 100 ng/ml TLR1/2 agonist, Pam3Cys. The study was approved by the ethics committee of West China Hospital, Sichuan University, Chengdu, China. Written informed consent was obtained from all participants.

### Reverse transcription and quantification PCR

At 4 h post-stimulation, total RNA was isolated using an RNeasy mini kit (Qiagen, Dusseldorf, Germany) from the Pam3Cys-treated and untreated groups. cDNA was synthesized using the ReverTra Ace quantitative polymerase chain reaction (qPCR) kit (FSQ-101; Toyobo, Kagoshima, Japan). The reverse transcription conditions were 65°C for 5 min, followed by 37°C for 15 min and 98°C for 5 min.

qPCR was performed using RealMaster Mix (SYBR Green; FP202; Tiangen, Beijing, China). The qPCR was performed in an iCycler iQTM Optical Module (Beckman Coulter, Fullerton, CA, USA) under the following conditions: One cycle at 95°C for 30 sec, then 40 cycles at 95°C for 30 sec, 58°C for 30 sec and 72°C for 30 sec, followed by a melt curve from 55 to 95°C in 0.5°C increments and 10-sec intervals. The primers used are listed in Table I. All tests were conducted three times.

### Antibody array

Conditioned media from TLR1/2-treated and untreated PBLs were analyzed for protein expression using RayBio Human Antibody Array C Series 1000 (RayBiotech; Norcross, GA, USA), according to the manufacturer’s instructions. Blots were analyzed with ImageJ software (National Institutes of Health, Bethesda, MD, USA).

### Western-blot analysis

Proteins of PBLs were extracted using a standard mammalian protein extraction reagent (Pierce, Rockford, IL, USA) containing protease inhibitor (Roche Applied Science, Indianapolis, IN, USA). Lysates were clarified by centrifugation at 13,000 × g for 10 min at 4°C. Protein concentration was measured using a Micro BCA Protein Assay kit (Pierce). The total protein at 20 μg was loaded on 15% SDS-polyacrylamide gels and transferred to nitrocellulose membranes (Invitrogen, Carlsbad, CA, USA). The membranes were blocked with 5% skimmed dried milk in Tris-buffered saline (TBS) containing 0.2% Tween-20 (TBST) for 1 h at room temperature, and then incubated at 4°C overnight with the primary antibodies (Table II). Next, the membranes were washed in TBST (3 times, 60 min) and incubated with secondary antibody conjugated to horseradish peroxidase (1:5000; Abcam, Cambridge, UK) for 1 h at room temperature. Antigen-antibody complexes were visualized using X-ray film following exposure to enhanced chemiluminescence reagent (Amersham Biosciences, Fairfield, CT, USA). The gray analysis of western blotting was completed using ImageJ software (National Institutes of Health).

### Data analysis

The qPCR data were analyzed using Bio-Rad iQ5 software. Glyceraldehyde 3-phosphate dehydrogenase was used as an internal control. Normal PBLs were used as a negative control. Results were expressed as the mean ± standard error of the mean using SPSS 16.0 (IBM, SPSS Statistics, Armonk, NY, USA). Values of P<0.05 and P<0.001 were considered to indicate a significant difference compared with the control group. The figures were completed using GraphPad Prism5 (GraphPad Software, Inc., LA Jolla, CA, USA).

## Results

### Detection of cytokine/chemokine secretion in supernatant

**Figure 1:**
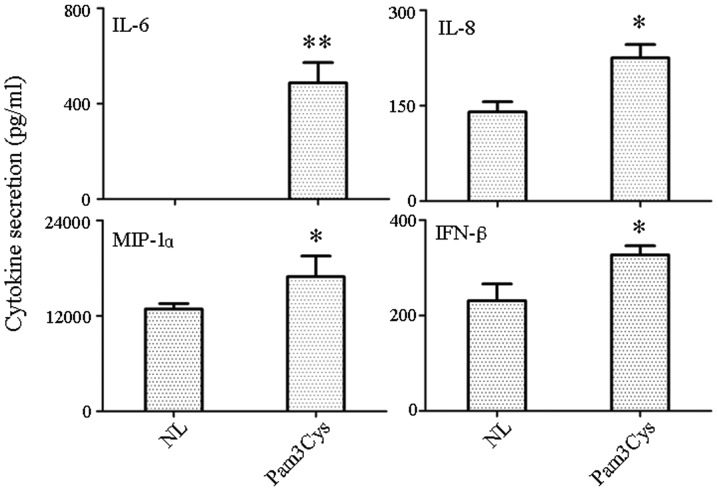
Cytokine/chemokine secretion in supernatant. ^**^P<0.001 and ^*^P <0.05 versus control group. TLR1, Toll-like recepor 1; NL, normal control; Pam3Cys, TLR1 agonist-treated; IL, interleukin; MIP-1α, macrophage inflammatory protein; IFN, interferon.

Supernatant from TLR1/2 agonist-treated and untreated PBLs was tested for the presence of secreted chemokines and cytokines by RayBio antibody-chip assays. A total of 20 molecules were chosen for detection [α-fetoprotein (AFP), albumin, E-Selectin, intracellular adhesion molecule 1 (ICAM-1), interferon (IFN)-α and -γ, interleukin (IL)-10, -12, -18, -1β, -4, -5, -6 and -8, monocyte chemoattractant protein (MCP)-1 and -3, macrophage inflammatory protein (MIP)-1α, Notch-1, transforming growth factor β and vascular endothelial growth factor]. The antibody-chip assay indicated that the TLR1/2 agonist resulted in the increased secretion of IL-6, IL-8, MIP-1α and IFN-β (Fig. 1). The most significant increase was in IL-6 (P<0.001), while the other three molecules (IL-8, MIP-1α and IFN-β) were not increased as significantly as IL-6 (P<0.05). Other target genes either did not result in detectable protein levels or had protein levels that remained the same.

### Quantification survey of immunomodulatory genes by qPCR

Cytokine/chemokine expression variations were analyzed by qPCR in an effort to identify candidate genes responsible for TLR1/2 agonist-mediated changes in PBLs. Therefore, changes in the expression of these genes, either due to microenvironment or pathogen stimulation, could greatly affect the biological function of PBLs.

**Figure 2:**
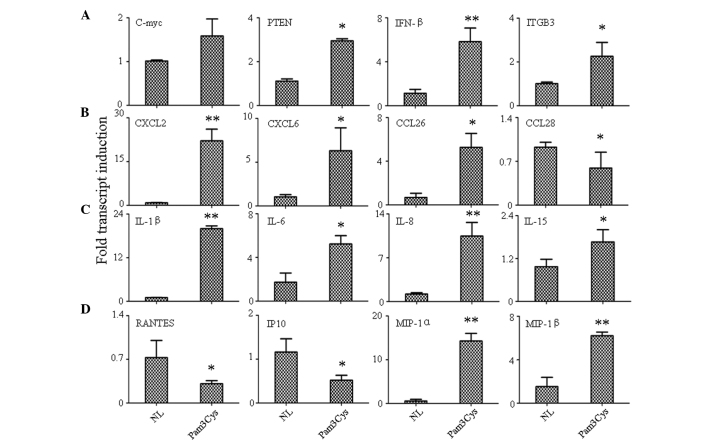
Detection of gene expression variation by qPCR. (A) Cancer related genes, (B) chemokines, (C) interleukins and (D) growth factors. ^**^P<0.001 and ^*^P<0.05 versus control group. qPCR, quantitative polymerase chain reaction; NL, normal control; Pam3Cys, TLR1 agonist-treated; PTEN, phosphatase and tensin homolog; IFN, interferon; ITGB3, integrin β3; CXCL, chemokine (C-X-C motif) ligand; CCL, chemokine (C-C motif) ligand; IL, interleukin; RANTES, regulated upon activation, normal T cell expressed and secreted; IP-10, interferon γ-induced protein 10; MIP, macrophage inflammatory protein.

Four tumor-related genes were found to be expressed: Phosphatase and tensin homolog (PTEN), a well-known tumor suppressor; c-myc, an oncogene; ITGB3, an adhesion molecule; and IFN-β, a defense factor. Activation of TLR1/2 increased the expression of all selected genes (P<0.05); the most significant increase was detected in INF-β (P<0.001; Fig. 2A).

In chemokine detection, the result indicated that the TLR1 agonist could increase the expression of chemokine (C-X-C motif) ligand 2 (CXCL2; P<0.001), CXCL6 (P<0.05) and chemokine (C-C motif) ligand 26 (CCL26; P<0.05), while the expression of CCL28 (P<0.05) was downregulated by Pam3Cys (Fig. 2B). The expression of other chemokines, including CCL21, CCL25, CXCL2 and CXCL3 remained the same along with the normal control (data not shown). In IL detection, it was found that all tested ILs were either upregulated, including IL-1β (P<0.001), IL-6 (P<0.05), IL-8 (P<0.001) and IL-15 (P<0.05) (Fig. 2C) or remained unchanged (IL-2, IL7, IL-9 and IL-18) when stimulated by the TLR1/2 agonist. Finally, growth factor detection was analyzed and the result showed that the expression of regulated upon activation, normal T cell expressed and secreted (RANTES; P<0.05) and interferon γ-induced protein 10 (IP-10; P<0.05) were increased, while MIP-1α (P<0.001) and MIP-1β (P<0.001) were inhibited by TLR1/2 stimulation (Fig. 2D).

### TLR1/2 agonist activates the downstream signal kinase

**Figure 3:**
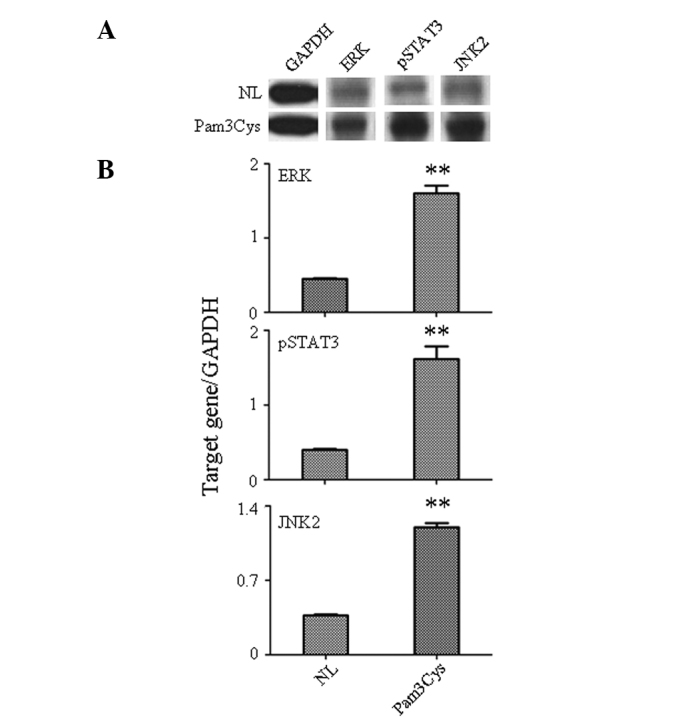
Analysis of kinase signal protein expression by western blotting. (A) Western blotting result; (B) gray analysis of western blotting. ^**^P<0.001; ^*^P<0.05 versus control group. NL, normal control; Pam3Cys, TLR1 agonist-treated; GAPDH, glyceraldehyde 3-phosphate dehydrogenase; ERK, extracellular signal-related kinase; pSTAT3, phosphorylated signal transducer and activator of transcription 3; JNK2, c-Jun N-terminal kinase 2.

Activation of downstream signaling molecules following Pam3Cys stimulation of PBLs was assessed by western-blot analysis (Fig. 3). Levels of phosphorylated signal transducer and activator of transcription 3 (pSTAT3), c-Jun N-terminal kinase 2 (JNK2) and extracellular signal-related kinase (ERK) were analyzed in the Pam3Cys-treated PBLs due to their significant role in the control of cell apoptosis, differentiation, migration and proliferation. The western-blot analysis indicated the increased expression of pSTAT3, JNK2 and ERK (Fig. 3A) stimulated by Pam3Cys. The gray analysis also confirmed that the protein level of pSTAT3 (P<0.001), JNK2 (P<0.001) and ERK (P<0.001) was significantly increased in the Pam3Cys-treated PBLs compared with the untreated group (Fig. 3B). Since pSTAT3 is mostly activated by IL-6 stimulation (6), this indicates that the TLR1/2 agonist, through increase in the expression of IL-6, plays an important role in immune response function of PBLs. This result also confirmed that ERK and JNK were important in the IL-1β-, IL-8- and MIP-1α-mediated immune response in PBLs caused by Pam3Cys stimulation (7,8).

## Discussion

TLR1 and 2 play an important role in detecting Gram-positive bacteria, and are involved in the recognition of a variety of microbial components such as lipoproteins (9). The current study examined the expressional variation of immunomodulatory molecules of PBL stimulated by the TLR1/2 agonist. The technique of *in vitro* stimulation of human PBLs with TLR agonists followed by quantification of cytokine expression is not novel. This approach can be used to identify the PBLs immunological signature and to understand the TLR signaling pathways. In particular, alterations in the expression of genes implicated in the following biological processes were analyzed: i) tumor-related genes, ii) chemokines, iii) interleukins, iv) growth factors.

Although a former study showed that the TLR1/2 agonist increased the release of IL-8 and TNF-α (10), the present results demonstrated that activation of TLR1/2 could induce expression of numerous immunomodulatory factors, including tumor-related genes (c-myc, PTEN, IFN-β and ITGB3), chemokines (CXCL2, CXCL and CCL26), ILs (IL-1β, IL-6, IL-8 and IL-15) and growth factors (MIP-1α and MIP-1β), and only three factors showed decreased expression (CCL28, RANTES and IP10). However in antibody-chip assays of supernatant, only four factors showed expressional variation (IL-6, IL-8, MIP-1α and IFN-β). The explanation for this was either that the increase in gene expression was not equal with the increase in protein level or that the treatment time was too short (4 h) to detect the late expression of numerous factors in the culture supernatant. The study also uncovered the fact that at least three kinase signal proteins (pSTAT3, JNK2 and ERK) were significantly induced by the TLR1/2 agonist, which indicated that there were a number of kinase signal pathways involved in the immune response that were induced by activation of the TLR1/2 ligand.

The key difference between the present study and the previous literature is that the present study surveyed more factors that were significant in promoting the pro-inflammatory status, while in the majority of other studies, only a few cytokines were detected. This difference may miss the complexity of the TLR1/2 response, including the increased expression of c-myc, PTEN and the CXCLs that was observed in Pam3Cys, which had not been reported previously.

This study examined a broader range of molecules, which were significant in immune modulation. The limitation of the study was that there was only one time-point (4 h) detected; the treatment time of TLR1/2 should therefore be extended, as certain later response molecules will fail to be detected. Based on this study, the enhanced TLR1/2-induced release of pro-inflammatory conditions by PBLs indicates a possible dysregulation in the innate immune system. Our further studies will extend the treatment time of the TLR1/2 agonist and broaden the signal pathway assay.

## Figures and Tables

**Table I:** List of primers for qPCR analysis.

Gene	Forward primer	Reverse primer	GenBank number
c-myc	CAAGACTCCAGCGCCTTCTC	GTTGAGTAACGAGCTGACCCC	AM393287
CXCL2	AGGTGAAGTCCCCCGGAC	GCCCATTCTTGAGTGTGGCT	NM_002089
CXCL6	GCTGAGAGTAAACCCCAAAACG	GGAGCACTGCGGGCC	NM_002993
CCL26	CCAAGACCTGCTGCTTCCAA	GAATTCATAGCTTCGCACCCA	NM_006072
CCL28	CTCGCCATCGTGGCCTT	GCAATGGGAAGTATGGCTTCTG	AF220210
IFN-β	CAGCAATTTTCAGTGTCAGAAGCT	TCATCCTGTCCTTGAGGCAGT	M28622
IL-1β	ACGAATCTCCGACCACCACT	CCATGGCCACAACAACTGAC	M15330
IL-6	GACCCAACCACAAATGCCA	GTCATGTCCTGCAGCCACTG	M14584
IL-8	CTGGCCGTGGCTCTCTTG	CCTTGGCAAAACTGCACCTT	NM_000584
IL-15	GACCCCACCAAAGCTGGAC	TCACAGTGCTGCTGTCTGCTG	M90391
IP-10	TGAAATTATTCCTGCAAGCCAA	CAGACATCTCTTCTCACCCTTCTTT	NM_001565
ITGB3	TGCCGCCCTGCTCATCTGGA	TCCTGCAATCGTGGCACAGGC	NM_000212
MIP-1α	AGCTGACTACTTTGAGACGAGCAG	CGGCTTCGCTTGGTTAGGA	NM_002983
MIP-1β	CTGCTCTCCAGCGCTCTCA	GTAAGAAAAGCAGCAGGCGG	NM_002984
RANTES	GACACCACACCCTGCTGCT	TACTCCTTGATGTGGGCACG	NM_002985
PTEN	ACCATAACCCACCACAGC	CAGTTCGTCCCTTTCCAG	NM_058074
GAPDH	GAAGGTGAAGGTCGGAGTC	GAAGATGGTGATGGGATTTC	J04038

qPCR, quantitative polymerase chain reaction; CXCL, chemokine (C-X-C motif) ligand; CCL, chemokine (C-C motif) ligand; IFN, interferon; IL, interleukin; IP-10, interferon γ-induced protein 10; ITGB3, integrin β3; MIP, macrophage inflammatory protein; RANTES, regulated upon activation, normal T cell expressed and secreted; PTEN, phosphatase and tensin homolog; GAPDH, glyceraldehyde 3-phosphate dehydrogenase.

**Table II:** Primary antibodies for western blotting.

Name	Company	Catalog number	Molecular weight, kDa
JNK2	Abcam	2037-1	54
ERK	Abcam	1171-1	44
pSTAT3	Abcam	2236-1	92
GAPDH	Abcam	ab8245	37

JNK, c-Jun N-terminal kinase; ERK, extracellular signal-regulated kinase; pSTAT, phosphorylated signal transducer and activator of transcription 3; GADPH, glyceraldehyde 3-phosphate dehydrogenase.
